# Supplementary material for: The choline transporter Slc44a2 controls platelet activation and thrombosis by regulating mitochondrial function
Source: Nat Commun. 2020 Jul 13;11:3479. doi: 10.1038/s41467-020-17254-w (PMC7359028; doi:10.1038/s41467-020-17254-w)
Supplement: Supplementary file 1 — Supplementary Information [file 41467_2020_17254_MOESM1_ESM.pdf]

Supplementary Information

The choline transporter Slc44a2 controls platelet activation and thrombosis by regulating mitochondrial function.

Lowenstein et al.

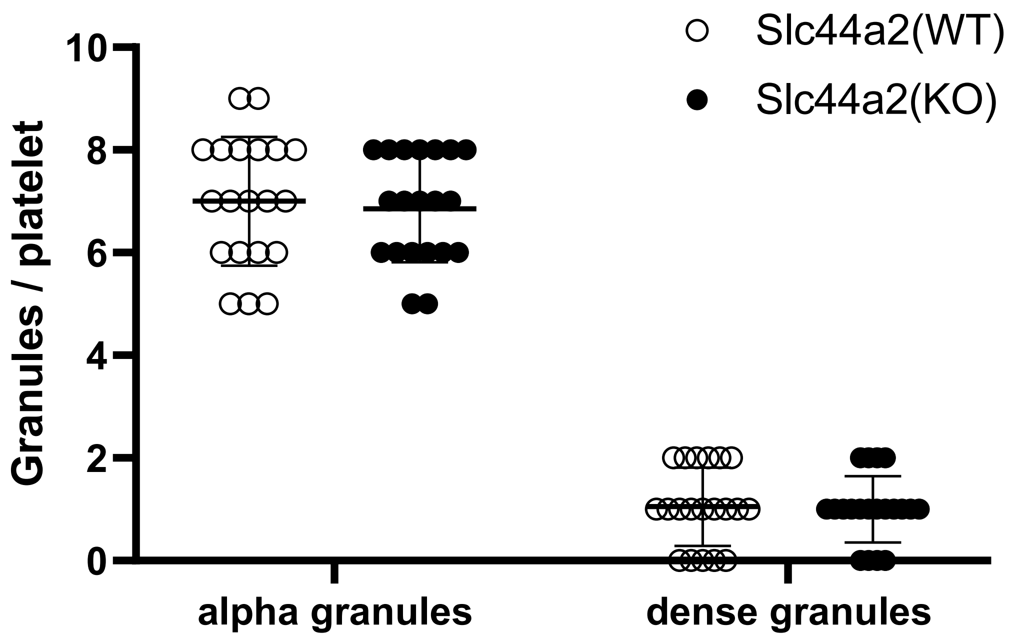

Supplementary Figure 1. Alpha-granule and dense granule count per platelet for Slc44a2(WT) and Slc44a2(KO) mice. Platelets were harvested from mice, fixed, and imaged by electron microscopy. The number of alpha-granules and dense granules per platelet were counted. There are no significant differences in the number of granule between wild-type and knockout mouse platelets (n = 21 platelets ± S.D.).

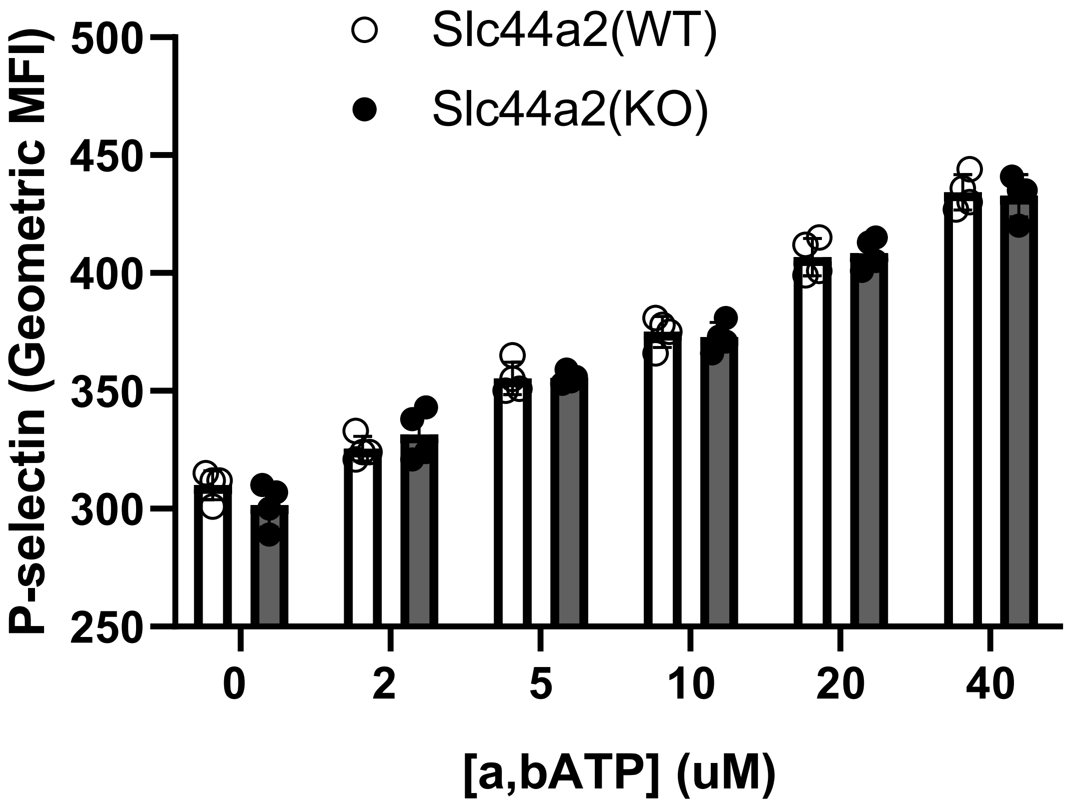

Supplementary Figure 2. Platelets from mice were treated with a non-hydrolysable ATP analog (alpha, beta, ATP) and the externalization of P-selectin was measured by flow cytometry. There is no difference in stimulation between platelets from Slc44a2(WT) and Slc44a2(KO) mice (n = 3 mice ± S.D.).

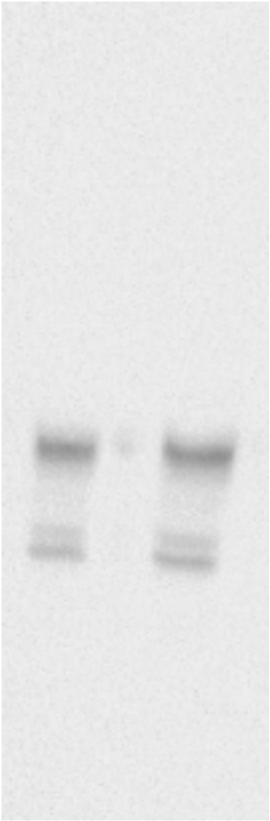

Immunoblot for Slc44a2

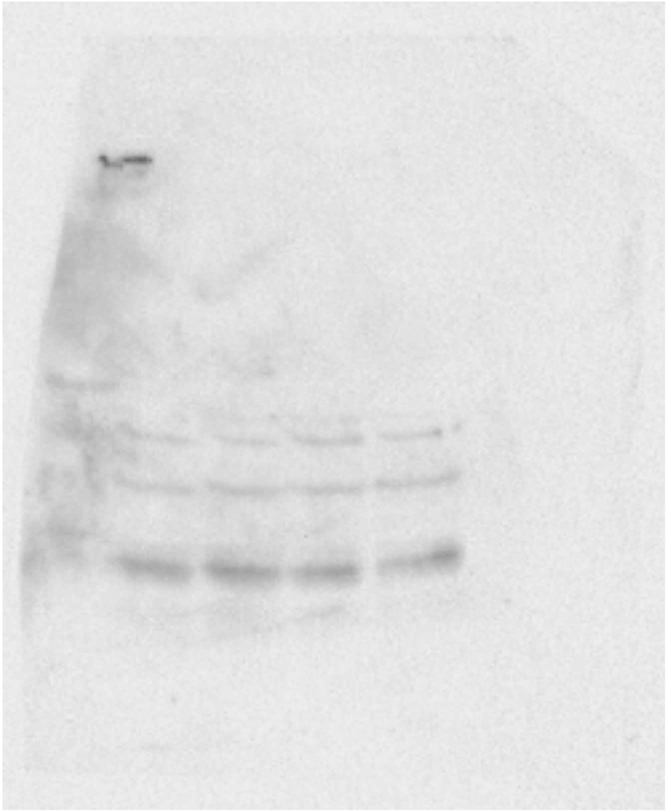

Immunoblot for Gapdh

Supplementary Figure 3. Platelets express Slc44a2. Immunoblot of Slc44a2 and GAPDH for Figure 1.

CBC parameter	WT	KO	P-value
WBC	10.94 ± 0.98	6.69 ± 0.46	0.002
MONO	0.153 ± 0.03	0.123 ± 0.08	0.55
NEUT	0.79 ± 0.26	0.62 ± 0.12	0.34
LYMPH	9.98 ± 1.09	5.95 ± 0.40	0.004
RBC	9.81 ± 0.08	9.51 ± 0.10	0.02
PLT	666.67 ± 57.62	615.33 ± 66.31	0.39

Supplementary Table 1. Complete blood counts from blood from Slc44a2(WT) and Slc44a2(KO) mice (n = 3 mice ± S.D. representative of 3 separate experiments).

1,5-AG/1-deoxyglucose	5-MTHF	Cholic acid	Glutamic acid	Inosine	NADP+	Palmitic acid	Taurochenodeoxycholic acid
11(12)-EET	5-Oxo-ETE	Citraconic acid	Glutathione disulfide	Inosine-15N4	NADPH	Pantothenic acid	Taurocholic acid
12-Oxo-ETE	8(9)-EET	Citric acid/Isocitric acid	Glutathione_reduced	IP3	N-Arachidonoyl Dopamine	Phe-d8	Thymidine
14(15)-EET	Acetoacetic acid	Citrulline	Glyceraldehyde-3-P / DHAP	Keto-isocaproic acid KIC / Keto-methylvalerate KMV	N-Arachidonoyl Taurine	Phosphocreatine	Thymine-d4
15-Oxo-ETE	Acetyl-CoA	Citrulline-d7	Glyceric acid	Kynurenic acid	N-Docosanoyl Taurine	Phosphoenolpyruvic acid	TMP (dTMP)
20-Hydroxy-N-Arachidonoyl Taurine	Aconitic acid	cyclic-AMP	Glycerol-3-phosphate	Kynurenine	Nicotinic acid	PIP2	UDP
2-Aminoadipic acid	Adenine	Deoxycholic acid	Glycochenodeoxycholic acid	Lactic acid	N-Lignoceroyl Taurine	PIP3	UDP-GlcNAc
2-Deoxyuridine	ADP	Deoxyuridine	Glycocholic acid	Lactose	N-Nervonoyl Taurine	Pseudouridine	UDP-glucose / -galactose
2-Hydroxybutaric acid	a-Ketoglutaric acid	D-Gluconic acid	Glyoxylic acid	Linoleic acid	N-Oleoyl Dopamine	Pyroglutamic acid	UDP-glucuronic acid
2-Hydroxyglutaric acid	Allantoin	DHA	GTP	L-Malic acid	N-Oleoyl Taurine	Pyruvic acid	UMP
2-ketoisovaleric acid	AMP	DHAP / glyceraldehyde-3-P	HETE	Malonic acid	N-Palmitoyl Taurine	Quinolinic acid	Uracil
2-Oxoadipic acid	Anthranilic acid	Dihomo-Gammi-Linolenoyl Ethanolamide	Hippuric acid	Mevalonic acid	Oleamide	Saccharopine	Uric acid
3-Aminoisobutyric acid	Arachidonic acid	dUMP	Homogentisic acid	N-Acetyl-D-glucosamine	Oleoyl Glycine	S-Adenosyl-L-homocysteine	Uridine
3-Hydroxy-3-methylglutaryl-CoA	Arachindonoyl Phenylalanine	EPA	Hydroxyphenylpyruvic acid	N-Acetyl-L-Alanine	Oleoyl Leucine	Sorbitol	UTP / CTP / TTP
3-Hydroxybutyric acid	Aspartic acid	F1P / F6P / G1P	Hypoxanthine	N-Acetyl-L-Aspartic acid	Oleoyl Phenylalanine	Stearic acid	Xanthine
3-Hydroxykynurenine	ATP	FAD+	IMP	N-Acetyl-L-Glutamic acid	Oleoyl Serine	Succinic acid / Methylmalonic acid	Xanthosine
3-Phosphoglyceric acid	Bilirubin	Fructose-1,6-P	Indole-3-carboxylic acid	N-Acetyl-L-Glutamine	Orotic acid	Sucrose	Xanthurenic acid
5(6)-EET	Butyric acid	Fumaric acid	Indole-3-lactic acid	N-Acetyl-L-Methionine	Oxalic acid	T3	
5-HIAA	CDP	G6P	Indole-3-propanoic acid	NAD+	Oxaloacetic acid	T4	
5-Methyluridine	CDP-Choline (citicholine)	Glucose/Fructose/Galactose_waterloss	Indoxyl-sulfate	NADH_reduced	Oxypurinol	Taurine	

Supplementary Table 2. List of metabolites for metabolomic analyses of platelets.

Metabolite	Fold change	- log(P)	Metabolite	Fold change	- log(P)	Metabolite	Fold change	- log(P)	Metabolite	Fold change	- log(P)
1,5-AG/1-deoxyglucose	1.16	0.14	Cholic acid	0.85	0.44	Inosine	1.80	2.76	Palmitic acid	0.60	0.73
11(12)-EET	0.46	1.09	Citraconic acid	5.95	1.56	Inosine-15N4	1.31	0.79	Pantothenic acid	1.09	0.09
12-Oxo-ETE	0.54	1.18	Citric acid/Isocitric acid	1.06	0.23	IP3	0.00	0.00	Phe-d8	1.40	0.68
14(15)-EET	0.58	0.62	Citrulline	1.11	0.22	Keto-isocaproic acid KIC / Keto-methylvalerate KMV	5.95	1.56	Phosphocreatine	0.00	0.00
15-Oxo-ETE	0.15	0.98	Citrulline-d7	1.10	0.12	Kynurenic acid	4.80	3.37	Phosphoenolpyruvic acid	0.60	0.21
20-Hydroxy-N-Arachidonoyl Taurine	0.98	0.02	cyclic-AMP	0.91	0.12	Kynurenine	2.35	0.91	PIP2	0.00	0.00
2-Aminoadipic acid	1.16	0.77	Deoxycholic acid	1.02	0.02	Lactic acid	0.85	0.28	PIP3	0.00	0.00
2-Deoxyuridine	1.30	0.91	Deoxyuridine	1.26	0.80	Lactose	1.21	0.11	Pseudouridine	0.75	0.39
2-Hydroxybutaric acid	0.80	0.16	D-Gluconic acid	1.49	0.92	Linoleic acid	0.91	0.60	Pyroglutamic acid	0.92	0.16
2-Hydroxyglutaric acid	1.50	0.57	DHA	0.86	0.38	L-Malic acid	1.41	0.51	Pyruvic acid	0.80	0.12
2-ketoisovaleric acid	1.30	0.42	DHAP / glyceraldehyde-3-P	1.10	0.14	Malonic acid	0.94	0.43	Quinolinic acid	2.05	0.63
2-Oxoadipic acid	1.60	0.63	Dihomo-Gammi-Linolenoyl Ethanolamide	0.94	0.12	Mevalonic acid	2.32	1.53	Saccharopine	1.63	0.60
3-Aminoisobutyric acid	0.67	0.65	dUMP	0.00	0.00	N-Acetyl-D-glucosamine	0.40	0.91	S-Adenosyl-L-homocysteine	1.10	0.20
3-Hydroxy-3-methylglutaryl-CoA	0.00	0.00	EPA	1.17	0.67	N-Acetyl-L-Alanine	0.82	0.34	Sorbitol	0.95	0.15
3-Hydroxybutyric acid	0.42	0.68	F1P / F6P / G1P	1.19	0.25	N-Acetyl-L-Aspartic acid	1.17	0.32	Stearic acid	0.94	0.13
3-Hydroxykynurenine	0.98	0.05	FAD+	0.00	0.00	N-Acetyl-L-Glutamic acid	2.50	0.96	Succinic acid / Methylmalonic acid	0.51	2.25
3-Phosphoglyceric acid	0.73	0.26	Fructose-1,6-P	0.93	0.46	N-Acetyl-L-Glutamine	0.85	0.27	Sucrose	0.84	0.10
5(6)-EET	0.45	0.88	Fumaric acid	1.25	0.58	N-Acetyl-L-Methionine	2.06	1.83	T3	1.09	0.14
5-HIAA	1.38	2.81	G6P	1.19	0.26	NAD+	0.00	0.00	T4	1.40	0.39
5-Methyluridine	0.76	0.31	Glucose/Fructose/Galactose _waterloss	1.19	1.28	NADH_reduced	0.00	0.00	Taurine	1.08	0.18
5-MTHF	7.75	2.53	Glutamic acid	1.08	0.14	NADP+	0.00	0.00	Taurochenodeoxycholic acid / Taurochenodeoxycholic acid	1.86	2.03
5-Oxo-ETE	0.75	0.48	Glutathione disulfide	1.60	0.68	NADPH	0.00	0.00	Taurocholic acid	2.40	1.71
8(9)-EET	0.51	1.28	Glutathione_reduced	6.85	2.67	N-Arachidonoyl Dopamine	0.00	0.00	Thymidine	1.17	0.12
Acetoacetic acid	3.55	1.80	Glyceraldehyde-3-P / DHAP	0.76	0.70	N-Arachidonoyl Taurine	1.36	0.48	Thymine-d4	1.22	0.54
Acetyl-CoA	0.00	0.00	Glyceric acid	1.62	0.68	N-Docosanoyl Taurine	0.93	0.45	TMP (dTMP)	1.15	0.37
Aconitic acid	1.94	1.36	Glycerol-3-phosphate	0.57	1.29	Nicotinic acid	0.61	0.35	UDP	0.49	1.13
Adenine	0.63	1.00	Glycochenodeoxycholic acid	2.35	1.32	N-Lignoceroyl Taurine	0.96	0.05	UDP-GlcNAc	0.71	0.31
ADP	0.80	0.27	Glycocholic acid	1.63	0.71	N-Nervonoyl Taurine	0.83	0.41	UDP-glucose / -galactose	0.89	0.10
a-Ketoglutaric acid	1.34	0.62	Glyoxylic acid	4.02	2.47	N-Oleoyl Dopamine	0.00	0.00	UDP-glucuronic acid	1.04	0.01
Allantoin	1.27	0.15	GTP	0.70	0.42	N-Oleoyl Taurine	1.15	0.31	UMP	0.00	0.00
AMP	0.82	0.37	HETE	0.53	1.62	N-Palmitoyl Taurine	0.89	0.18	Uracil	0.51	1.23
Anthranilic acid	0.00	0.00	Hippuric acid	2.53	2.36	Oleamide	1.55	0.65	Uric acid	0.47	1.68
Arachidonic acid	1.35	1.04	Homogentisic acid	2.65	2.70	Oleoyl Glycine	0.00	0.00	Uridine	0.66	1.33
Arachindonoyl Phenylalanine	0.54	0.31	Hydroxyphenylpyruvic acid	2.81	2.29	Oleoyl Leucine	0.56	0.48	UTP / CTP / TTP	0.42	0.80
Aspartic acid	0.92	0.61	Hypoxanthine	0.83	0.62	Oleoyl Phenylalanine	0.00	0.00	Xanthine	0.46	1.24
ATP	0.68	0.31	IMP	1.30	0.64	Oleoyl Serine	1.31	0.18	Xanthosine	0.37	1.67
Bilirubin	0.00	0.00	Indole-3-carboxylic acid	1.48	0.49	Orotic acid	0.84	0.23	Xanthurenic acid	1.79	0.61
Butyric acid	1.25	0.83	Indole-3-lactic acid	2.63	1.87	Oxalic acid	0.77	0.30			
CDP	0.44	0.65	Indole-3-propanoic acid	1.67	2.00	Oxaloacetic acid	0.91	0.15			
CDP-Choline (citicholine)	0.00	0.00	Indoxyl-sulfate	1.38	0.30	Oxypurinol	0.00	0.00			

Supplementary Table 3. Metabolomic Data. Lysates were prepared from platelets from 3 WT and 2 KO mice and analyzed for metabolites as described in the methods section and shown in Figure 3.
