# Supplementary material for: The choline transporter Slc44a2 controls platelet activation and thrombosis by regulating mitochondrial function
Source: Nat Commun. 2020 Jul 13;11:3479. doi: 10.1038/s41467-020-17254-w (PMC7359028; doi:10.1038/s41467-020-17254-w)
Supplement: Supplementary file 3 — Reporting Summary [file 41467_2020_17254_MOESM3_ESM.pdf]

nature research

Corresponding author(s): Charles J. Lowenstein

Last updated by author(s): Jun 11, 2020

Reporting Summary

Nature Research wishes to improve the reproducibility of the work that we publish. This form provides structure for consistency and transparency in reporting. For further information on Nature Research policies, see our Editorial Policies and the Editorial Policy Checklist.

Statistics

For all statistical analyses, confirm that the following items are present in the figure legend, table legend, main text, or Methods section.

n/a	Confirmed	
☐	☒	The exact sample size (n) for each experimental group/condition, given as a discrete number and unit of measurement
☐	☒	A statement on whether measurements were taken from distinct samples or whether the same sample was measured repeatedly
☐	☒	The statistical test(s) used AND whether they are one- or two-sided<br>*Only common tests should be described solely by name; describe more complex techniques in the Methods section.*
☐	☒	A description of all covariates tested
☐	☒	A description of any assumptions or corrections, such as tests of normality and adjustment for multiple comparisons
☐	☒	A full description of the statistical parameters including central tendency (e.g. means) or other basic estimates (e.g. regression coefficient) AND variation (e.g. standard deviation) or associated estimates of uncertainty (e.g. confidence intervals)
☐	☒	For null hypothesis testing, the test statistic (e.g. F, t, r) with confidence intervals, effect sizes, degrees of freedom and P value noted<br>*Give P values as exact values whenever suitable.*
☒	☐	For Bayesian analysis, information on the choice of priors and Markov chain Monte Carlo settings
☒	☐	For hierarchical and complex designs, identification of the appropriate level for tests and full reporting of outcomes
☒	☐	Estimates of effect sizes (e.g. Cohen's d, Pearson's r), indicating how they were calculated

Our web collection on statistics for biologists contains articles on many of the points above.

Software and code

Policy information about availability of computer code

Data collection	N/A
Data analysis	N/A

For manuscripts utilizing custom algorithms or software that are central to the research but not yet described in published literature, software must be made available to editors and reviewers. We strongly encourage code deposition in a community repository (e.g. GitHub). See the Nature Research guidelines for submitting code & software for further information.

Data

Policy information about availability of data

All manuscripts must include a data availability statement. This statement should provide the following information, where applicable:
- Accession codes, unique identifiers, or web links for publicly available datasets
- A list of figures that have associated raw data
- A description of any restrictions on data availability

All data are freely available upon request.

Field-specific reporting

Life sciences study design

All studies must disclose on these points even when the disclosure is negative.

Sample size	Sample size was determined based on preliminary data and power analysis.
Data exclusions	All data were tested to identify outliers defined by > 2 SD. No outliers which would be appropriate for exclusion were detected, so no data were excluded.
Replication	Data reported are representative of multiple experiments.
Randomization	n/a. We used mice of known genotype.
Blinding	Our approach (numeric labeling of samples) means investigators are blinded to genotypes.

Reporting for specific materials, systems and methods

We require information from authors about some types of materials, experimental systems and methods used in many studies. Here, indicate whether each material, system or method listed is relevant to your study. If you are not sure if a list item applies to your research, read the appropriate section before selecting a response.

Materials & experimental systems

n/a	Involved in the study	
☐	☒	Antibodies
☐	☒	Eukaryotic cell lines
☒	☐	Palaeontology and archaeology
☐	☒	Animals and other organisms
☒	☐	Human research participants
☒	☐	Clinical data
☒	☐	Dual use research of concern

Methods

n/a	Involved in the study	
☒	☐	ChIP-seq
☐	☒	Flow cytometry
☒	☐	MRI-based neuroimaging

Antibodies

Antibodies used	All antibodies, vendors, fluor and clone information are provided in Materials and Methods.
Validation	Antibodies were used at manufacturer recommended concentrations, and validated in house at the respective manufactu

Eukaryotic cell lines

Policy information about cell lines

Cell line source(s)	HEK293 cells from a collaborators lab
Authentication	None of the cell lines were authenticated.
Mycoplasma contamination	The cell lines were not tested for mycoplasm
Commonly misidentified lines (See ICLAC register)	HEK293 were used as a positive control for Slc44a2.

Animals and other organisms

Policy information about studies involving animals; ARRIVE guidelines recommended for reporting animal research

Laboratory animals	male mice mus musculus 6-8 wks old from the Jackson laboratories
Wild animals	N/A
Field-collected samples	N/A
Ethics oversight	All in vivo procedures and usage of mice were performed by protocols which were approved by the Division of Laboratory Animal Medicine (DLAM) at the University of Rochester Medical Center.

Note that full information on the approval of the study protocol must also be provided in the manuscript.

Flow Cytometry

Plots

Confirm that:

- [x] The axis labels state the marker and fluorochrome used (e.g. CD4-FITC).
- [x] The axis scales are clearly visible. Include numbers along axes only for bottom left plot of group (a 'group' is an analysis of identical markers).
- [x] All plots are contour plots with outliers or pseudocolor plots.
- [x] A numerical value for number of cells or percentage (with statistics) is provided.

Methodology

Sample preparation	Materials and Methods contains all descriptions for sample preperation
Instrument	BD LSRII / Accuri C6
Software	Flowjo was used for all data analysis, FACS Diva was used for all data acquisition.
Cell population abundance	N/A We did not use cell sorting.
Gating strategy	We analyzed the entire population for mean fluorescence intensity. No gates were applied.

- [ ] Tick this box to confirm that a figure exemplifying the gating strategy is provided in the Supplementary Information.
